# Dr. Thomas Tobin

**Published:** 1917-04

**Authors:** 


**Dr. Thomas Tobin, Buffalo** 1882, of Tyrone, Pa., died Feb. 13, of cerebral haemhorrhage, while making a professional call.

